# The effects of female sex hormones on the human cornea across a woman’s life cycle

**DOI:** 10.1186/s12886-023-03085-y

**Published:** 2023-08-16

**Authors:** Donel S. Kelly, Sabhyta Sabharwal, David J. Ramsey, Melina I. Morkin

**Affiliations:** 1 United States Naval Hospital Okinawa, Ginowan, Japan; 2 Scheie Eye Institute, Philadelphia, PA 19104 USA; 3 Department of Ophthalmology, Lahey Hospital & Medical Center, Burlington, MA 01805 USA; 4 Department of Ophthalmology, Tufts University School of Medicine, 800 Washington St, Boston, MA 02111 USA

**Keywords:** Female hormones, Menstruation, Pregnancy, Menopause, Cornea

## Abstract

The cornea is a hormone-responsive tissue that responds to changing levels of female sex hormones. This review focuses on the structural and functional changes in the human cornea associated with the hormonal milestones of menarche, pregnancy, and menopause, as well as consequences stemming from the use of exogenous sex hormones for fertility control and replacement. Articles were identified by searching PubMed without language or region restrictions. The primary outcomes evaluated were changes in central corneal thickness (CCT), intraocular pressure (IOP), and quality of the ocular tear film. The potential impact of hormone-associated changes on the diagnosis and surgical management of common eye diseases, as well as the potential use of sex hormones as therapeutic agents is also considered. Understanding the physiological effects of female sex hormones on the cornea is important because that knowledge can shape the management decisions physicians and women face about ocular health across their life stages.

## Introduction

**Fig. 1:**
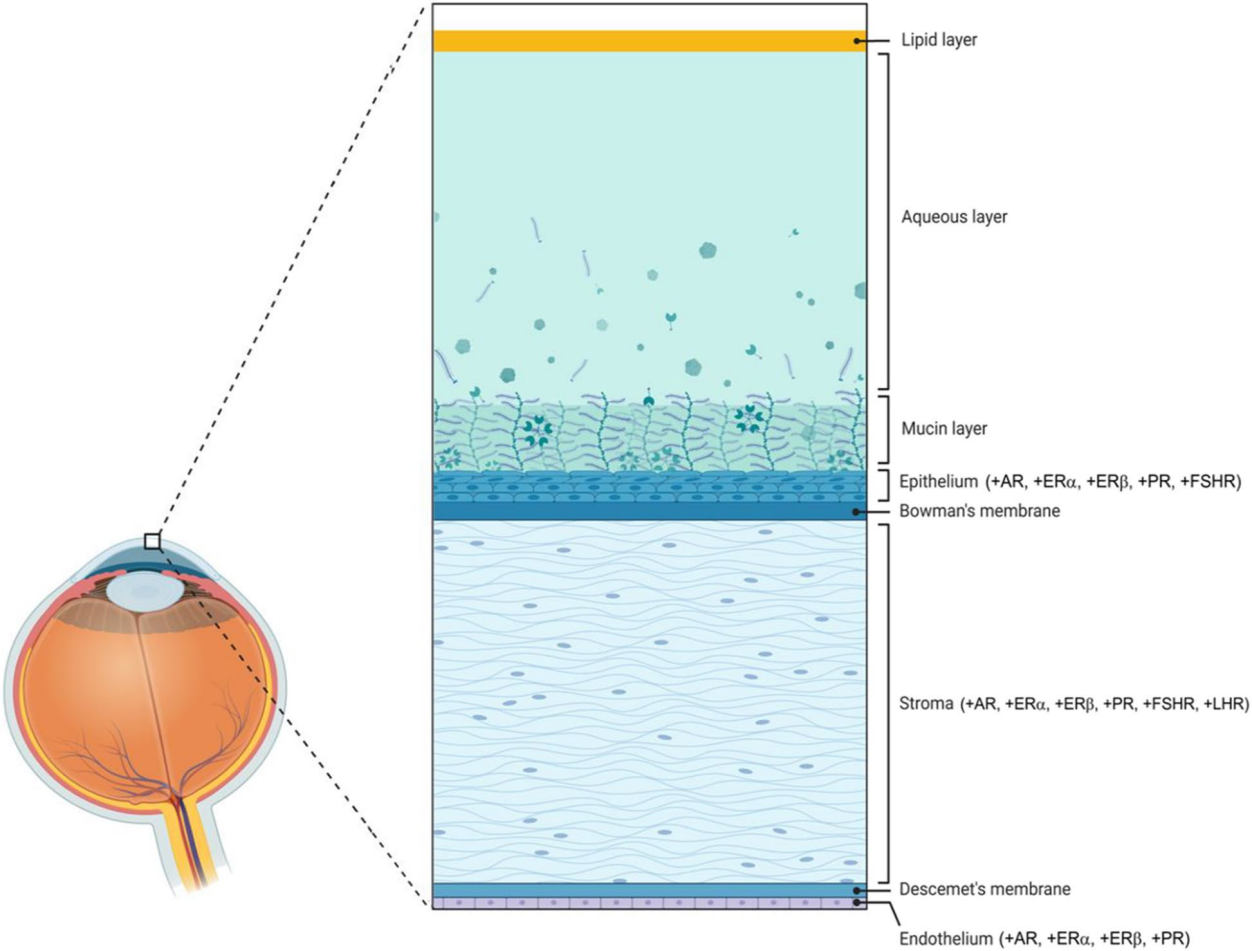
Diagram illustrating the layers of the human cornea and location of hormonal receptors. Notations: AR (androgen receptor); ER (estrogen receptor); PR (progesterone receptor); LHR (luteinizing hormone receptor); FSHR (follicle-stimulating hormone receptor). A plus-sign ( +) indicates evidence of receptor or enzyme mRNA expression in that respective layer. Figure was made in ©BioRender-biorender.com

Sex hormones play an essential role in the development and function of the human cornea [1]. The cornea is a gender-dependent and hormone-responsive tissue that expresses sex hormone receptors in at least three of the five corneal layers, including the corneal epithelium, stroma, and endothelium (Fig. 1) [1, 2]. The tissues that comprise the anterior segment, including the cornea, also express enzymes capable of endogenous sex hormone production [3]. In addition to endogenous expression, the ocular tear film overlying the corneal surface allows hormones and other signaling molecules to reach the cornea by means of diffusion [1]. Sex hormones produced or released from intraocular sources, including the retina and vitreous, may also access the cornea through direct diffusion or by means of the internal ocular circulation of aqueous fluid [4, 5].

The balance of sex hormones varies across a woman’s life and has downstream effects on central corneal thickness (CCT), intraocular pressure (IOP), and the ocular tear film [6–19]. The mechanisms by which sex hormones govern these corneal features and related physiologic processes remain under active investigation, but are likely related to a combination of direct effects on the tissues themselves, as well as indirect effects through their actions on neuroendocrine, immune, and vascular regulation [3].

**Fig. 2:**
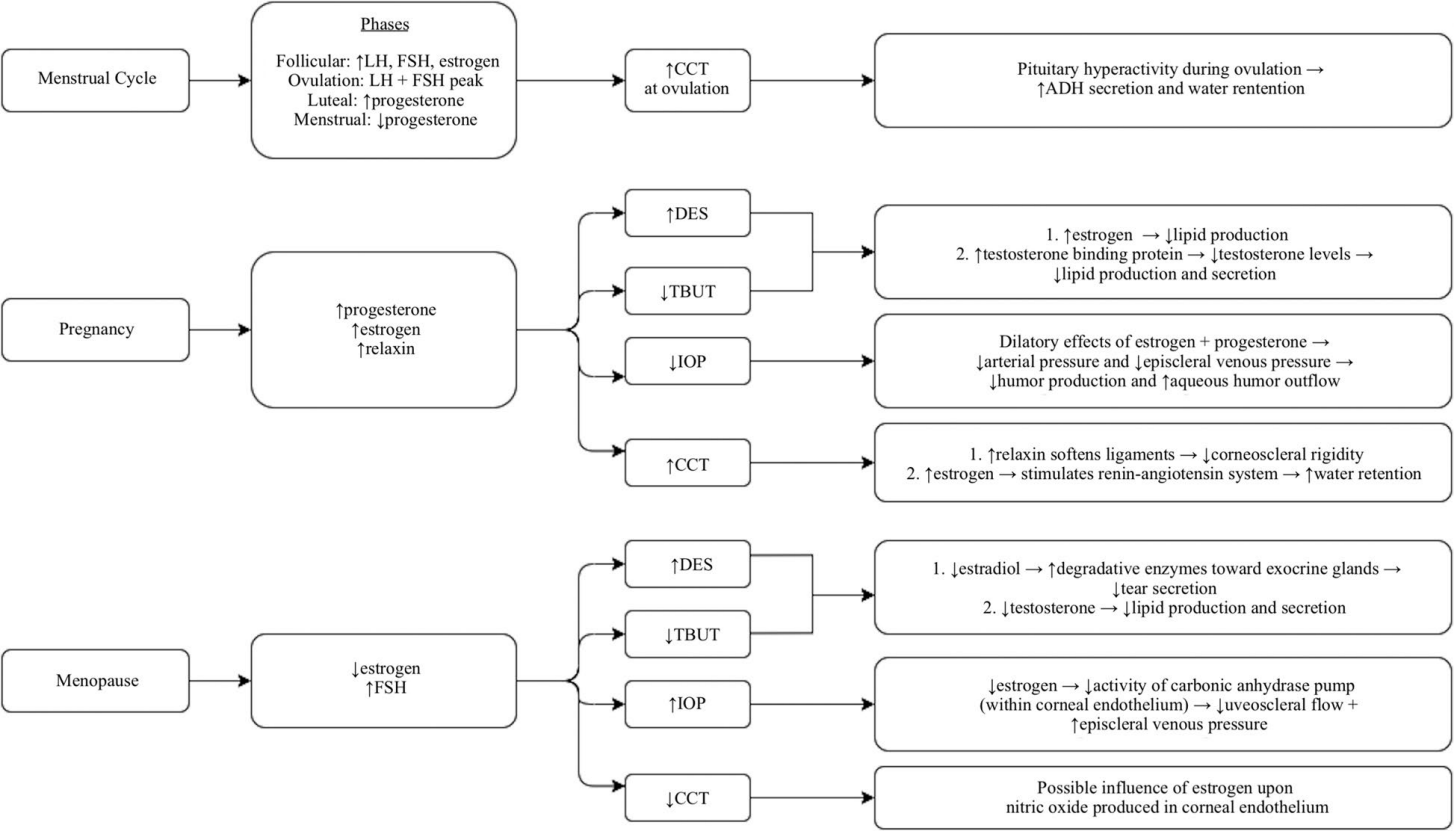
Schematic diagram of hormonal changes across the different stages of a woman’s life, illustrating their impact on IOP, CCT, TBUT, and DES, as well as the proposed etiologies for each of these changes. Notations: CCT (central corneal thickness); IOP (intraocular pressure); TBUT – Tear Break-Up Time; DES – Dry Eye Symptoms; LH – Luteinizing Hormone; FSH – Follicle-Stimulating Hormone; ↑—increase; ↓—decrease; →—subsequently

This review evaluates the physiologic impact of female sex hormones on the structure and function of the human cornea associated with the milestones of menarche, pregnancy, and menopause (Fig. 2). It also takes into account the consequences of exogenous sex hormones used for fertility control and replacement, and the potential of these agents to treat corneal disease. Finally, the implications of those hormonal changes on the clinical and preoperative evaluation for refractive and glaucoma management are considered. It is important to understand the physiological effects of female sex hormones on the structure and function of the cornea because that knowledge helps to shape management decisions about ocular health that physicians and women face across their life stages.

## Methods

This systematic review was conducted according to PRISMA guidelines. A comprehensive literature search of the MEDLINE and Embase databases was performed using combinations of the following search terms: “cornea” and “women,” "menstrual cycle,” “pregnancy,” “menopause," or "hormone replacement therapy.” Articles were identified without language or region restrictions. Reference lists from each of the articles retrieved were also scrutinized to identify further relevant studies. The primary outcomes identified included changes in CCT, IOP, tear break-up time (TBUT), ocular surface disease index (OSDI), and Schirmer’s test caused by changing levels of female sex hormones associated with the milestones of menarche, pregnancy, and menopause. Articles that considered the potential impact of each of those hormone-associated changes on the preoperative evaluation for ophthalmic surgery, as well as the potential use of sex hormones or their derivatives as therapeutic agents, were reviewed. Finally, articles that failed to report original data were excluded in favor of primary sources. The primary search yielded over 680 references. The studies were divided between D.K. and S.S. for independent reviews of inclusion criteria described above. In cases of discrepancy in opinion, the article was reviewed by a third author. Title, abstract, and full article reviews derived 55 articles appropriate for inclusion in this review.

### Corneal changes at menarche and during the menstrual cycle

#### Variations in hormonal levels

The follicular phase begins on day one of the menstrual cycle and is characterized by the onset of menstruation and continues until ovulation. Estrogen is produced by ovarian follicles activated by the rising levels of follicle-stimulating hormone (FSH) and luteinizing hormone (LH). Most active follicles undergo atresia, typically leaving just one to mature. Once estrogen levels reach a critical point, positive-feedback modulation leads to an LH surge, which allows the mature follicle to rupture and, in the process, release an egg from the ovary for possible fertilization. This process, known as ovulation, typically occurs around day 14 of a 28-day cycle. The luteal phase, typically spanning from day 14 to 28, begins with the development of the corpus luteum from the ovarian follicle and ends with pregnancy or the abolition of the corpus luteum. If fertilization does not occur, the corpus luteum becomes the corpus albicans, and levels of progesterone and estrogen fall, thus stimulating FSH to recruit follicles for the next follicular phase [20]. Estrogen and progesterone levels differ significantly during the follicular, ovulatory, and luteal phases of the menstrual cycle. Estrogen levels are lowest at the follicular phase and highest at ovulation. Progesterone levels are lowest at the follicular phase and most elevated at the luteal phase [6].

#### Intraocular pressure

IOP is both a risk factor for the development of glaucoma and a biomarker utilized in the diagnosis and treatment of the disease. Age, race, gender, and sex hormones are among the factors that govern the levels of IOP [7]. These factors may influence the rate of aqueous production, the function of outflow pathways through the trabecular meshwork, related structures, and episcleral venous system, and corneal curvature and thickness [7]. Notably, fluctuations in female sex hormones have been linked to changes in IOP. In a study by Goldich et al. that included 22 women, IOP measured by Goldmann applanation tonometry increased at ovulation compared with the end of the menstrual cycle, with a difference of around 1 mmHg (*p* = 0.05) [8]. Although these changes may be associated with the fluctuations in female sex hormones, Mishra et al. suggested that the concurrent surge in systemic antidiuretic hormone (ADH) levels produced by the pituitary gland around ovulation might also contribute to the thickness of the cornea by increasing tissue hydration [9].

#### Corneal thickness

CCT is an important ocular parameter relevant to the management of many ocular conditions. CCT is intimately related to IOP measurement because thicker corneas tend to cause higher IOP readings using standard clinical measurement techniques [10]. Assessment of CCT is also necessary to establish whether sufficient corneal tissue is present for refractive surgery, such as laser-assisted in situ keratomileusis (LASIK) [21]. Hormone-related changes in CCT are therefore important to consider.

In an analysis of 105 women, Mishra et al. demonstrated that the central cornea tends to be thinnest at the start of the menstrual cycle (542 ± 4.21 um) and thickest at mid-cycle (i.e., during ovulation; 559 ± 4.50 um, *p* < 0.001) [9]. Several smaller studies have reported similar results [8]. A study by Ghahfarokhi et al. found that CCT was also greatest at ovulation (556 ± 7.11 um) and thinnest at the end of the cycle (536 ± 12.83 um, *p* < 0.001), rather than at the beginning of menstruation [11].

#### Ocular surface

The tear film plays a vital role in the function of the human cornea. It forms a protective and supportive barrier that conveys signaling molecules and provides both oxygen and nutritional support [22]. Hormonal changes regulate the makeup and balance of the tear film components [6, 12]. Dry eye disease (DED), a multifactorial disease of the ocular surface, is a prevalent cause of ocular discomfort and frequent clinic visits [13, 23, 24]. Measuring TBUT as a sign of tear film stability, Cavdar et al. found no significant changes in TBUT at any phase during the menstrual cycles of 17 premenopausal females [6]. This finding was also reflected by the lack of changes in the Schirmer I test, which evaluates tear production, across the phases of the menstrual cycle [6]. In another study conducted in 15 women without a history of preexisting dry eye symptoms, Versura et al. found a statistically significant difference in tear stability between menstruation and the luteal phase (*p *= 0.03) as well as between the follicular and luteal phases (*p* = 0.001), with the lowest TBUT in the follicular phase (11.1 ± 2.4 s) and highest in the luteal phase (13.4 ± 1.8 s) [12]. By contrast, tear stability among the 14 women with dry eye symptoms did not significantly change between phases.

In relation to symptoms related to dry eye disease, numerous studies have indicated that variations in symptoms may be associated with fluctuations in sex hormone levels during the menstrual cycle, particularly worsening around the time of peak estrogen levels [6, 12]. More specifically, Cavdar et al. demonstrated a significantly lower ocular surface disease index (OSDI) score during the follicular phase compared with both the ovulatory (*p* = 0.004) and luteal phases (*p* = 0.01) of the menstrual cycle [6]. These results were in accordance with a study by Nkiru et al. conducted in pregnant women, which shared the finding that symptom scores were lowest when estrogen and progesterone levels were decreased [25]. While TBUT and Schirmer I test results did not change significantly among phases of the menstrual cycle in the study by Cavdar et al., a significant relationship was shown between the above tests and OSDI at day 12 (ovulatory phase), indicating a possible correlation between subjective symptomatology and objective testing [6]. Versura et al. also reported similar symptom results in women with and without preexisting dry eye disease, with the highest OSDI scores in the luteal phase and lowest during menstruation [12].

### Corneal changes during pregnancy

#### Variations in hormonal levels

After fertilization, the corpus luteum is sustained by human chorionic gonadotropin (hCG) produced by the placenta [20, 26]. The corpus luteum releases estrogen and high progesterone levels until approximately 10 weeks, when the developing placenta is capable of producing adequate hormone levels to support pregnancy [26]. These hormone levels rise markedly during pregnancy and average more than 15 times the levels found in the non-gravid population [27].

#### Intraocular pressure

Fluctuations in female sex hormones during pregnancy have also been linked to changes in IOP [7, 10, 14, 28]. A meta-analysis by Wang et al. identified 15 studies that reported a significant reduction in IOP during the second and third trimesters of pregnancy compared with a non-gravid control group [7]. Subsequently, several additional studies have reported results in concordance with this meta-analysis. In a study comparing 165 pregnant with 105 non-pregnant women, the mean IOP was statistically lower in the pregnant cohort (13.2 ± 2.2 mmHg vs. 14.2 ± 2.7 mmHg, *p* = 0.001) [15]. In another study which included 235 women monitored across each of the trimesters of pregnancy and into the puerperal period, Tolunay et al. observed a significant, steady decline in IOP when comparing the first with the second and third trimesters of pregnancy, and a rebound in the postpartum period to levels indistinguishable from where they started in the first trimester of pregnancy [14]. Similarly, in a study by Efe et al. that performed a longitudinal evaluation of IOP in 25 women over the course of pregnancy found that IOPs decreased by an average of 9.5%, with the highest values in the first trimester and the lowest in the third trimester (13.8 ± 2.1 vs. 12.4 ± 2.08 mmHg, *p* < 0.001) [10]. IOP regressed to a level similar to that obtained in the first-trimester by 3-month postpartum. Finally, Naderan et al. reported a decrease in Goldmann and corneal-compensated IOP measurements during pregnancy in women with keratoconus, which persisted six months after pregnancy [28]. Bear in mind, the authors describe keratoconus to be a disorder of corneal elasticity, resulting in corneal thinning and thus suggesting a lower-pressure phenomenon.

As discussed by Wang et al., a widely proposed mechanism to account for the decrease in IOP observed during pregnancy hypothesizes a relationship between female sex hormones and an increase in aqueous humor outflow capacity, similarly to other body systems where venous capacity expands during pregnancy. Peak levels of progesterone, estrogen, and relaxin at the end of pregnancy correlate inversely with IOP [8]. Possible mechanisms to explain this change include the dilatory effects of progesterone and estrogen, resulting in decreased arterial pressure, subsequent reduction in humor production, and increased humor outflow facilitated by reduced episcleral venous pressure [7, 14]. It is well described that estradiol increases nitric oxide-induced relaxation of the trabecular meshwork [14, 16], while progesterone competes with endogenous glucocorticoid receptor sites, thus inhibiting the ocular hypertensive effect of glucocorticoid [7, 14, 15, 28]. Another proposed mechanism attributes the decrease in IOP to increased relaxin levels during pregnancy, softening ligaments and thus reducing corneoscleral rigidity [7, 10, 14].

#### Corneal thickness

Efe et al. have identified a synchronous relationship between IOP and CCT changes across the trimesters of pregnancy [10]. When comparing the first and third trimesters, a 3.1% increase in CCT corresponded to the 9.5% decrease in IOP discussed above (*p* < 0.001). Efe et al. also found CCT measurements in the second (567 ± 22 µm) and third trimesters (574 ± 24 µm) to be significantly higher when compared with the first trimester (561 ± 22.17 µm) and 3 months postpartum (562 ± 23 µm, *p* < 0.001) [10]. Likewise, Wang et al. noted a significant increase in CCT during the second trimester compared with the non-pregnant control group [7]. In contrast, Naderan et al. found a steady decrease in the mean CCT when comparing before pregnancy, during pregnancy, and 6 months postpartum (458 ± 42, 439 ± 47, and 431 ± 48 µm, *p* < 0.001) [28]. Of note, this cohort study evaluated patients with keratoconus. When evaluating healthy eyes during pregnancy, another study by Naderan et al. reported that CCT did not significantly change during pregnancy [29].

Given the presence of estrogen, progesterone, and androgen receptors within corneal epithelial cells, it is plausible that changes in female sex hormone levels could directly lead to changes in CCT [30, 31]. As mentioned above, an alternative mechanism for the increased CCT in pregnancy includes estrogen-induced upregulation of the renin-aldosterone system resulting in systemic water retention [7, 10, 17, 32].

Additionally, the literature suggests that hormonal changes during pregnancy, including the increase in estrogen and relaxin levels, may result in a reduction of corneal stiffness and subsequent keratoconus (KC) progression [28]. The documented presence of estrogen receptors within human corneal keratocytes supports this potential mechanism [33].

The study by Naderan et al. on 11 patients with keratoconus revealed a substantial increase in KC severity during and after pregnancy compared with a non-pregnant control group using the Amsler-Krumeich classification for KC [28]. Similarly, a study by Bilgihan et al. reported four KC patients who had disease progression during pregnancy, three of whom manifested progression at the sixth gestational month [34]. It is worth noting that several studies failed to show a statistically significant difference in CH or CRF in healthy eyes during pregnancy [18, 29].

A retrospective analysis by Taneja et al. of 12 patients with keratectesia concluded that all patients experienced symptoms of vision deterioration in as little as two months up to one year after the onset of pregnancy [35]. Ten of these patients developed new onset post-refractive surgery keratectasia and two patients experienced an exacerbation of KC. This study is not the first to propose an association between pregnancy and the onset of post-LASIK ectasia. Hafezi et al. described a case of keratectasia diagnosis during pregnancy that recurred during a second pregnancy despite corneal collagen cross-linking (CXL) [36]. In this case report, a 33-year-old woman with no pre-existing corneal abnormalities had bilateral LASIK with corneal stability over a two-year follow-up period, followed by a significant decrease in visual acuity during the seventh gestational month and subsequent confirmation of keratectasia diagnosis. Despite disease regression following CXL in both eyes, the patient had progression of keratectasia noted during the sixth gestational month of her *second* pregnancy. In a follow-up letter to the editor by Hafezi et al., the group reported five patients who underwent LASIK and later developed progressive iatrogenic keratectasia during pregnancy despite corneal stability documented for years prior, with the latest onset occurring during a pregnancy that started nine years after LASIK [37].

#### Ocular surface

Tear film changes during pregnancy may impact the function of the ocular surface. Several studies have found increased ocular discomfort and decreased tear production in pregnant women [15, 23, 25]. In a cohort of 201 pregnant women, Asiedu et al. found the prevalence of dry eye disease to be 40.8% [23]. Measuring OSDI among 134 pregnant women, Nkiru et al. found the highest OSDI score in the third trimester [25]. Both the OSDI score and prevalence of DED were significantly higher in the third trimester compared to the second trimester and postpartum periods. In a study by Ibraheem and coresearchers on 165 pregnant and 105 non-pregnant women, the average TBUT was significantly lower among the pregnant patients (37.0 ± 1.7 vs. 50.1 ± 19.1 s, *p* < 0.001) [15]. However, the average Schirmer’s reading was significantly higher in pregnant women compared with their non-pregnant counterparts (25.0 ± 9.3 mm vs. 22.1 ± 10.8 mm, *p* = 0.018) [15]. The authors attributed these results to the majority of pregnant patients being in early gestation and thus not yet at maximum hormonal upregulation.

Several mechanisms are postulated to exacerbate DED in pregnancy. There may be direct damage to lacrimal acinar cells through pregnancy-augmented immune reactivity of prolactin [23, 38]. Other proposed DED-related processes include estrogen-induced acinar cell death [15, 38] and dehydration secondary to nausea, vomiting, and effects of medications used to manage these symptoms [38]. It is thought that testosterone results in the synthesis and secretion of lipids from meibomian glands, while estrogen causes a decrease in lipid production, thereby increasing the risk of dry eye [24]. The pro-secretory activity of testosterone on meibomian glands is antagonized by sharing receptors with progesterone and by decreased testosterone levels secondary to increased production of testosterone-binding protein [15].

### Corneal changes during Menopause

#### Variations in hormonal levels

The hormonal shifts in menopause follow a pattern opposite to those in pregnancy. While hormone levels rise during pregnancy, menopause is characterized by a drop in both estrogen and progesterone levels [16, 31]. Similarly, the corneal changes commonly noted during menopause are typically the opposite of those associated with pregnancy, further supporting the proposition that these effects are related to changing levels of female sex hormones.

#### Intraocular pressure

After menopause, IOP tends to rise. Panchami et al. reported significantly higher average IOPs in 60 postmenopausal women compared to 60 premenopausal women (18.5 mmHg vs. 15.2 mmHg, *p* < 0.05) [16]. Similarly, in a study by Birgul et al. comparing 153 premenopausal women with 142 postmenopausal women, the authors measured significantly higher average IOPs in the postmenopausal eyes (17.1 ± 1.7 vs. 15.7 ± 2.7 mmHg, *p* < 0.001) [19]. The mechanism by which female sex hormones may lower IOP was discussed above in the context of pregnancy. In menopause, as estrogen and progesterone levels fall, the effect would be the opposite. Birgul et al. suggested that the IOP increase is secondary to the reduction in estrogen specifically [19]. The hypothesis presented is that estrogen reduction hinders the function of the carbonic anhydrase pump located in the corneal endothelium, causing a decrease in uveoscleral flow, increase in episcleral venous pressure, and subsequent rise in ocular tension. Finally, the decreased estrogen levels in menopause may contribute to the steepening of the horizontal curvature in postmenopausal women, which could not only impact the patient’s refraction, but also the measured IOP [31].

#### Corneal thickness

CCT tends to decrease after menopause. In a prospective, case–control, single-blinded study, Keskin et al. measured CCT in 40 premenopausal and 40 postmenopausal women [39]. The study found significantly lower average CCT values in the postmenopausal group (521.2 ± 38 μm vs. 561 ± 42.8 μm, *p* < 0.005). The research team posited that the lack of estrogen reduced the production and release of nitric oxide from the corneal endothelium, leading to the reduction in corneal thickness. Furthermore, they suggested that the loss of estrogen-induced systemic water retention could similarly impact corneal thickness. Lastly, Sanchis-Gimeno et al. found that the CCT of 30 postmenopausal women with dry eye was significantly lower than that of 32 postmenopausal women without dry eye (533.1 + 4.7 μm vs. 547.6 + 15.1 μm, *p* < 0.001) [40]. The study ascribed these findings to increased evaporation of the tear film with subsequent rise in tear fluid osmolarity and thus ultimately decreased thickness [40].

#### Ocular surface

Several studies have suggested a higher incidence of DED among postmenopausal women [13, 24]. Garcia-Alfaro and coresearchers reported the prevalence of DED symptoms to be 76.4% versus 80.5% in perimenopausal and postmenopausal women, respectively (*p* = 0.029) [13]. Additionally, the average OSDI score was significantly higher in postmenopausal women (30.61 ± 19.97 vs. 26.81 ± 18.19, *p* < 0.001). Another study concluded tear volume and stability measured by Schirmer’s and TBUT tests, respectively, to be notably diminished in postmenopausal women compared with premenopausal women [41]. The literature suggests inflammation of the lacrimal gland, diminished meibomian gland tissue, and reduced lipid production secondary to androgen deficiency as the possible underlying mechanisms [42–45]. Nuzzi et al.’s clinical experience supports this current hypothesis as several cancer patients treated with anti-androgen drugs would report symptoms of ocular burning, photophobia, and foreign body sensation, consistent with DED [45]. Moreover, decreased estradiol triggers upregulation of degradative enzymes toward exocrine glands, and thus, reduced tear secretion [43]. The correlation between sex hormone levels and symptomatology of DED remains inconclusive [24, 43]. This is exemplified by the previously discussed increase in DED severity in pregnant and postmenopausal women and suggests other factors could be at play.

#### Hormonal therapies

The cornea, like other tissues influenced by female hormone levels, is also believed to respond to external hormonal treatments, such as hormone replacement therapy (HRT) and hormonal preparation for in-vitro fertilization (IVF). Dry eye symptoms are commonly reported by postmenopausal women, prompting several studies to investigate the impact of HRT on these symptoms, yielding varying results. HRT typically involves the administration of exogenous estrogens with the addition of progestins for women with an intact uterus. A recent meta-analysis of 17 studies found significant improvement in dry eye symptoms one month after systemic HRT use [46]. However, this improvement was no longer statistically significant by the three and six-month follow-up. When comparing DED symptoms in postmenopausal subjects with and without concurrent HRT use, the rate was significantly higher among those on HRT (odds ratio [OR] 1.69; 95% confidence interval [CI], 1.49–1.91 for HRT containing only estrogen, and OR 1.29; 95% CI, 1.13–1.48 for estrogen with progesterone/progestin) [47]. These data suggest an elevated risk of ocular discomfort associated with HRT use, most commonly with estrogen-only HRT [24, 47]. On the other hand, a review by Peck et al. discussed evidence indicating that prolonged duration of HRT eventually leads to decreased number of ocular complaints [24]. While future studies are needed to investigate the impact of systemic HRT on postmenopausal DED, others have begun exploring the clinical potential of local HRT in the form of topical estradiol ophthalmic formulations. However, one randomized, placebo-controlled trial failed to detect significant differences in Schirmer’s test scores using topical 17-β-estradiol-3-phosphate drops [48].

Corneal effects of IVF have also been studied among premenopausal women undergoing hormone-based reproductive assistance. The exogenous hormones involved in an IVF cycle may vary but often include some combination of FSH, hCG, gonadotropin-releasing hormone (GnRH) agonists, and LH, making it somewhat challenging to attribute ocular changes to a specific hormone. In a study by Parihar et al., which included 32 women undergoing IVF, no significant changes in IOP or CCT were noted, although these results may have been limited by the sample size [49]. By the third trimester, a statistically significant increase in dry eye disease in at least one eye, defined by a Schirmer I below 10 mm, was noted among patients receiving IVF, in agreement with the results discussed above in the pregnancy section. The observed reduction in tear secretions was partially explained by the previously noted mechanisms caused by changes in estrogen and progesterone levels, as well as decreased free testosterone. Without a proper control group, however, these findings should be analyzed with caution [49]. In another study, Colak et al. compared tear film parameters before and after ovulation induction, which led to significantly higher basal levels of estradiol (*p* < 0.001) [50]. They determined TBUT scores to be elevated after ovulation induction (6.2 ± 2.8 s vs. 8.4 ± 1.4 s, *p* = 0.001). In accordance, Schirmer’s test values were also significantly higher after ovulation (14.3 ± 7.1 mm vs. 20.6 ± 6.2 mm, *p* < 0.001). Differences in symptomatology using OSDI scores were not statistically significant [50].

In postmenopausal women, Coksuer et al. investigated the impact of systemic drospirenone and estradiol on tear film parameters and found substantially decreased values of Schirmer’s and TBUT tests before treatment compared to after treatment [51]. OSDI scores demonstrated an expected inverse correlation. Another study compared the efficacy of systemic tibolone, an HRT agent with estrogenic, progestogenic, and androgenic effects, and estradiol/medroxyprogesterone acetate on the ocular surface of postmenopausal women. Only those on HRT with tibolone exhibited markedly increased values of Schirmer’s (*p* < 0.001) and TBUT (*p* < 0.001) tests [42]. This improvement was primarily attributed to the androgenic activity of tibolone on the lacrimal gland and goblet cells as the estrogen-progesterone combination did not exhibit a similar impact [42]. Of note, the androgen receptor protein exists in the human cornea [52]. This supports the notion that androgen deficiency could be a major contributor to dry eye disease [42, 44, 48]. Along these lines and as alluded to above, the topical use of 17-β-estradiol-3-phosphate drops did not significantly change Schirmer’s test scores in postmenopausal women [48]. In a study in men with idiopathic hypogonadotropic hypogonadism, androgen replacement therapy had little effect on the cornea and tear function [53].

Based upon the understanding of the effect of female sex hormones on the cornea, several authors have gone on to speculate that these molecules or related compounds could be leveraged as potential therapeutic interventions. For example, Wei et al. postulated that estrogen might be used to protect retinal ganglion cells (RGCs) by virtue of its activation of collagen synthesis [54]. This may not only decrease the rigidity of the cornea (thereby decreasing the IOP), but could also enhance the compliance of other parts of the eye such as the lamina cribrosa, where such a change could protect RGC axons from compression.

Another potential area where female sex hormones have shown promise is their use in the treatment of refractive errors, although human clinical studies are presently lacking. Using a rabbit model, Leshno et al. discovered that topical application of estrogen eye drops resulted in a 0.6-diopter myopic shift that regressed with treatment cessation, supporting the notion that corneal refractive error can be modified pharmacologically through the direct action of sex hormones [55]. Whether systemic or topical estrogen supplementation beneficially impacts ocular surface health remains inconclusive [44, 48].

#### Impact of female sex hormone-associated changes on the preoperative evaluation for eye surgery

The available data suggests that pregnancy is a risk factor involved in the pathogenesis of corneal ectasia and the progression of pre-existing KC [28, 34, 35, 56]. If future studies supported its use, CXL could be more extensively considered to treat women with high-risk KC who contemplate future pregnancy [28, 34, 35]. Given that pregnancy may also stimulate post-LASIK ectasia, Hafezi et al. suggested that the preoperative counseling of LASIK should include mentioning the risk of ectasia in susceptible women of childbearing age [37]. Future research comparing photorefractive keratectomy (PRK) with LASIK may support a preference for surface ablation rather than LASIK in female patients with a history or prospective risk of hormonal imbalance, as suggested by Taneja et al. [35]. From experience, Nuzzi et al. has found it necessary at times to discontinue hormonal treatments prior to surgical management of refractive defects in order to achieve stable, satisfactory post-operative results [45].

While more extensive studies are needed to confirm the precise pattern of changes in CCT during the menstrual cycle, some studies have suggested statistically significant changes in CCT between the cycle’s different phases. Such variations can be critical in determining potential candidates or ideal timing for surgery [8, 9]. As shown by Goldich et al., an overestimation of the corneal thickness and an underestimation of hysteresis may occur if the patient is evaluated at the time of ovulation [8]. These changes in CCT during the menstrual cycle indicate that a menstrual history may be imperative before refractive surgery evaluation, contact lens fitting, or glaucoma assessment [9]. We propose that these evaluations include an assessment of a woman’s menstrual cycle phase. Also, the transient physiological changes in CCT and IOP during and after pregnancy need to be recognized when diagnosing or treating women for glaucoma [10], because if not taken into account they can lead to the implementation of unnecessary anti-glaucoma therapy or surgery, as well as increased patient anxiety [30]. Finally, although IOP generally remained within the normal range, the rise in IOP measured after menopause may influence the diagnosis or management of those women with glaucoma or suspect status [16].

## Conclusion

Female sex hormones impact the structure and function of the cornea throughout a woman’s life. The accumulating body of knowledge emphasizes the significance of understanding these effects in the management of corneal diseases and the potential for developing targeted therapeutics. Notably, the presence of sex hormone receptors in the cornea underscores its gender-dependent and hormone-responsive nature, opening avenues for innovative treatment strategies. However, to deepen our understanding, future studies with larger patient cohorts, appropriate controls, and refined laboratory models are warranted. Understanding the effects of sex hormones on the physiology of the cornea may help clinicians and their patients better understand the ocular symptoms women experience as they age, improve risk assessment when planning ocular surgery, and allow interventions and procedures to be tailored more precisely.

## Data Availability

All data generated or analyzed during this study are included in this published article.
